# Selenium Nanoparticle and Melatonin Treatments Improve Melon Seedling Growth by Regulating Carbohydrate and Polyamine

**DOI:** 10.3390/ijms25147830

**Published:** 2024-07-17

**Authors:** Lu Kang, Yujiao Jia, Yangliu Wu, Hejiang Liu, Duoyong Zhao, Yanjun Ju, Canping Pan, Jiefei Mao

**Affiliations:** 1 Key Laboratory of National Forestry and Grassland Administration on Pest Chemical Control and Innovation Center of Pesticide Research, College of Science, China Agricultural University, Beijing 100193, China; 96208zx@163.com (L.K.);; 2 State Key Laboratory of Desert and Oasis Ecology, Key Laboratory of Ecological Safety and Sustainable Development in Arid Lands, Xinjiang Institute of Ecology and Geography, Chinese Academy of Sciences, Urumqi 830011, China; 3 Institute of Agricultural Quality Standards and Testing Technology, Xinjiang Academy of Agricultural Sciences, Urumqi 830091, China; 4 School of Biological Science and Technology, University of Jinan, Jinan 250022, China

**Keywords:** bio-stimulants, melon, primary metabolism, secondary metabolism

## Abstract

Bio-stimulants, such as selenium nanoparticles and melatonin, regulate melon growth. However, the effects of individual and combined applications of selenium nanoparticles and melatonin on the growth of melon seedlings have not been reported. Here, two melon cultivars were sprayed with selenium nanoparticles, melatonin, and a combined treatment, and physiological and biochemical properties were analyzed. The independent applications of selenium nanoparticles, melatonin, and their combination had no significant effects on the plant heights and stem diameters of Jiashi and Huangmengcui melons. Compared with the controls, both selenium nanoparticle and melatonin treatments increased soluble sugars (6–63%) and sucrose (11–88%) levels, as well as the activity of sucrose phosphate synthase (171–237%) in melon leaves. The phenylalanine ammonia lyase (29–95%), trans cinnamate 4-hydroxylase (32–100%), and 4-coumaric acid CoA ligase (26–113%), as well as mRNA levels, also increased in the phenylpropanoid metabolism pathway. Combining the selenium nanoparticles and melatonin was more effective than either of the single treatments. In addition, the levels of superoxide dismutase (43–130%), catalase (14–43%), ascorbate peroxidase (44–79%), peroxidase (25–149%), and mRNA in melon leaves treated with combined selenium nanoparticles and melatonin were higher than in controls. The results contribute to our understanding of selenium nanoparticles and melatonin as bio-stimulants that improve the melon seedlings’ growth by regulating carbohydrate, polyamine, and antioxidant capacities.

## 1. Introduction

Melon (*Cucumis melo* L.) is a vital economic vegetable crop worldwide [1,2], with a global production of 27.3 million metric tons. For commercial cultivation, the cultivars Jiashi (JSG) and Huang mengcui (HMC) melon are the most important [3]. Selenium nanoparticles, melatonin, and other bio-stimulants have no negative effects on endophytic bacteria; therefore, they may be conducive to promoting plant health [4]. Plant primary metabolites are directly involved in growth, development, and reproduction [5]. Plant secondary metabolites are not only useful natural products, but they also play important roles in plant defense systems against pathogenic attacks and environmental stresses [6,7]. Plant secondary metabolites provide defense functions and regulate defense-signaling pathways that protect plants in response to herbivore invasion [8]. Plants produce three main types of secondary metabolites, phenols, terpenes, and nitrogen/sulfur compounds. The shikimic acid pathway leads to the formation of phenolic products involved in plant defenses. Terpenes, based on 5-C isoterpenoids, are toxins and deter herbivores, and nitrogen and sulfur compounds are synthesized mainly from amino acids [9].

Selenium nanoparticles are less toxic and more biocompatible than sodium selenate or sodium selenite [10]. Selenium nanoparticles increase the carboxylase activity of ribulose diphosphate and the chlorophyll content, especially through the activation of some key genes and proteins involved in the photosynthetic system. Treatments of 25–50 μmol·L^−1^ selenium nanoparticles reduce the large amount of reactive oxygen species (ROS) produced by nicotinamide adenine dinucleotide phosphate oxidase and enhance glutathione peroxidase (GSH-Px), thereby reducing protein carbonylation in rice seedlings [11]. Foliar applications of 25 mg·L^−1^ selenium nanoparticles increase cucumber height and leaf area [12]. The activities of antioxidant enzymes in plants are key factors in alleviating the effects of external stress [13,14]. The exogenous application of selenium nanoparticles improves the photosynthetic pigments of rape by increasing the activities of antioxidant enzymes, such as catalase (CAT), ascorbate peroxidase (APX), and superoxide dismutase (SOD). Additionally, the expression of stress-response genes enhances the drought and heat tolerances of rape [15,16].

Selenium nanoparticles enhance the antioxidant capacity mainly by improving the metabolic pathways of glutathione, carbon, and nitrogen, thereby improving various physiological indexes of maize that promote its growth [17]. Nanotechnology increases plant productivity, nutrient absorption, and agronomic soil properties, which result in improved plant growth and productivity [18,19,20]. Nanotechnology is widely considered to be on the cutting edge, with the potential to promote plant science research because nanoparticles have unique physicochemical properties compared with bulk particles [21,22].

Melatonin acts as a bio-stimulant of plant development, including germination, photosynthesis, and water utilization [23,24,25]. Melatonin is a key molecule in plant immune responses, along with nitric oxide, jasmonic acid, and salicylic acid [26,27]. Melatonin is involved in growth and photosynthetic processes. Melatonin causes multiple changes at the mRNA level and is a multi-regulatory molecule capable of coordinating many aspects of plant development [28]. Melatonin treatments significantly reduce hydrogen peroxide (H_2_O_2_) and malondialdehyde (MDA) contents, enhance the non-enzymatic antioxidant system’s capacity, and increase CAT, peroxidase (POD), SOD, and APX activity levels [29]. Melatonin enhances the antioxidant capacity of cotton, improves photosynthetic efficiency, reduces chlorophyll degradation and ROS accumulation, inhibits ABA synthesis, and delays the drought-induced senescence of cotton leaves [30]. Strawberries soaked in melatonin maintain fresh weights and fruit firmness, and they have reduced *Botrytis cinerea* infection levels. Additionally, melatonin treatments increase 1,1-diphenyl-2-picrohydrazyl radical (DPPH) clearance, as well as CAT, SOD, POD, and APX activity levels [31]. The application of melatonin improves the internal nutrition and flavor quality of tomato fruit by regulating the accumulations of primary and secondary metabolites during the ripening process [32].

Foliar applications of selenium nanoparticles enhance the antioxidant ability and the cucurbitacin B level of melon [33]. However, there have been no systematic reports on the effects of melatonin or selenium nanoparticle + melatonin combination applications on melon. This study aimed to explore the effects of selenium nanoparticle + melatonin combination treatments on the physiological and biochemical characteristics of melon plants. We investigated the effects of single applications of selenium nanoparticles and melatonin on plants, and we hypothesized that the combined application of bio-stimulant selenium nanoparticles + melatonin would have a synergistic effect. The goals were to provide a theoretical basis and technical support for the rational use of the selenium nanoparticle + melatonin combination to regulate the amino acid, carbohydrate, polyamine, and antioxidant capacities of melon.

## 2. Results

### 2.1. Effects of Selenium Nanoparticles and Melatonin on Plant Biomass

The effects of selenium nanoparticles, melatonin, and their combination on fresh and dry weights, plant heights, and stem diameters are shown in Figure S1. The independent applications of selenium nanoparticles and melatonin did not have significant effects on melon plant height or stem diameter. Compared with the controls, independent foliar spraying of selenium nanoparticles, melatonin, and selenium nanoparticles + melatonin increased the stem fresh weights of JSG by 23%, 6%, and 6%, respectively, and those of HMC by 9%, 6%, and 10%, respectively. All three treatments increased stem dry weights of JSG by 13–25% and of HMC by 8–19% compared with controls.

### 2.2. Lipoxygenase and Plant Hormones after Selenium Nanoparticle and Melatonin Treatments

**Figure 1:**
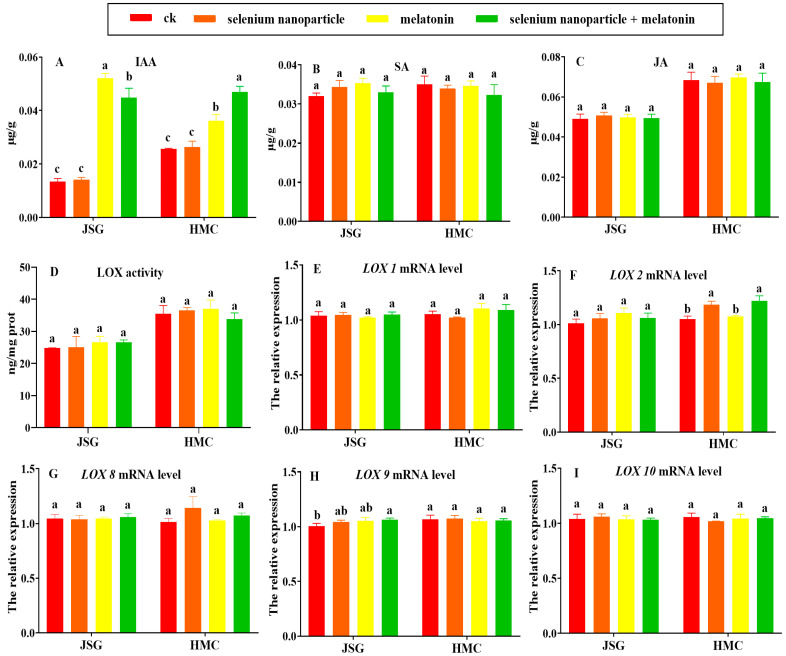
Effects of selenium nanoparticles and melatonin on plant hormone content and lipoxygenase in two melon cultivars. JSG and HMC refer to melon cultivars. Different letters indicate a significant difference (*p* < 0.05) between treatments. (**A**): IAA content, (**B**): SA content, (**C**): JA content, (**D**): LOX activity, (**E**): *LOX1* mRNA level, (**F**): *LOX2* mRNA level, (**G**): *LOX8* mRNA level, (**H**): *LOX9* mRNA level, (**I**): *LOX10* mRNA level.

The effects of selenium nanoparticles, melatonin, and the selenium nanoparticle + melatonin combination on plant hormones in melon are shown in Figure 1. Compared with controls, melatonin and selenium nanoparticles + melatonin significantly increased the IAA in leaves of JSG and HMC by 291–236% and 41–83%, respectively (Figure 1A). The three treatments had no significant effects on JA and SA (Figure 1B,C) in leaves of JSG and HMC compared with controls. The effects of selenium nanoparticles, melatonin, and the selenium nanoparticle + melatonin combination on lipoxygenase and mRNA levels in melon are shown in Figure 1D–I. The selenium nanoparticles, melatonin, and selenium nanoparticle + melatonin foliar interventions had no significant effects on LOX activity or the transcription levels in JSG and HMC leaves, except for *LOX2* and *LOX9* mRNA levels.

### 2.3. Effects of Selenium Nanoparticle and Melatonin Treatments on Amino Acid and Carbohydrate Metabolism

**Figure 2:**
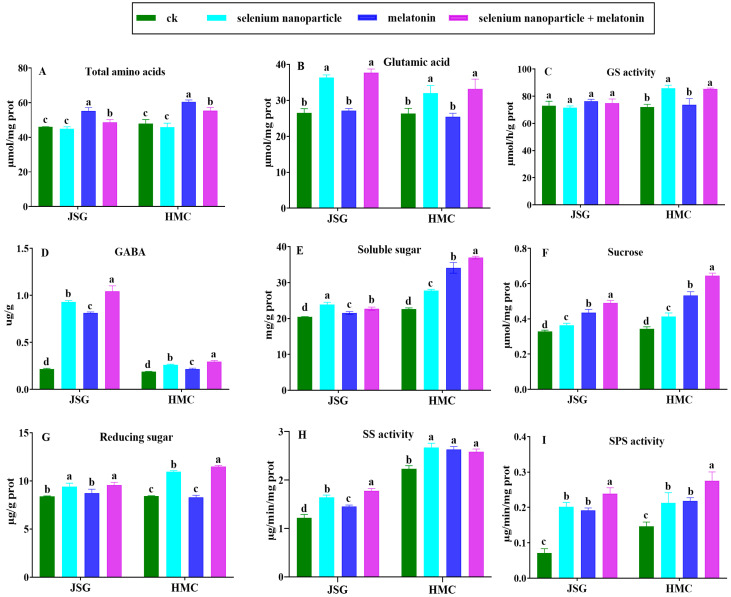
Effects of selenium nanoparticles and melatonin on amino acid content, carbohydrate metabolism in two melon cultivars. JSG and HMC refer to melon cultivars. Different letters indicate a significant difference (*p* < 0.05) between treatments. (**A**): Total amino acids content, (**B**): Glutamic acid content, (**C**): GS activity, (**D**): GABA content, (**E**): Soluble sugar content, (**F**): Sucrose content, (**G**): Reducing sugar content, (**H**): SS activity, (**I**): SPS activity.

The effects of selenium nanoparticles, melatonin, and the selenium nanoparticle + melatonin combination on amino acid and carbohydrate metabolism in melon leaves are shown in Figure 2. Compared with controls, melatonin and selenium nanoparticles + melatonin significantly increased the total amino acids of JSG, by 20% and 6%, respectively, and in HMC leaves by 26% and 16%, respectively (Figure 2A). The selenium nanoparticles and selenium nanoparticle + melatonin combination increased glutamate in leaves of JSG by 37% and 42%, respectively, and in HMC by 21% and 26%, respectively, compared with the controls (Figure 2B). The effects of the three treatments on the GS activity of JSG leaves were not significant. Compared with controls, the selenium nanoparticles and selenium nanoparticle + melatonin combination significantly increased the GS activity of HMC leaves, by 19% and 18%, respectively (Figure 2C). The three treatments significantly increased the GABA of JSG and HMC leaves by 279–386% and 16–57%, respectively, compared with controls (Figure 2D). Compared with controls, the three treatments significantly increased soluble sugars in the two cultivars by 6–63% (Figure 2E). The three treatments significantly increased the sucrose contents of the two cultivars by 11–88% compared with controls (Figure 2F). Compared with controls, the selenium nanoparticles and selenium nanoparticle + melatonin combination significantly increased the reducing sugars in JSG leaves by 12–14% and in HMC leaves by 30–36% (Figure 2G). The selenium nanoparticle, melatonin, and selenium nanoparticle + melatonin combination groups significantly increased SS activities in JSG leaves by 19–46% and in HMC leaves by 16–20% compared with controls (Figure 2H). The three treatments significantly increased the SPS activities in JSG leaves by 171–237% and in HMC leaves by 46–88% compared with controls (Figure 2I).

### 2.4. Effects of Selenium Nanoparticle and Melatonin Treatments on Secondary Metabolism

#### 2.4.1. Selenium Nanoparticle and Melatonin Effects on Lignin Synthesis

**Figure 3:**
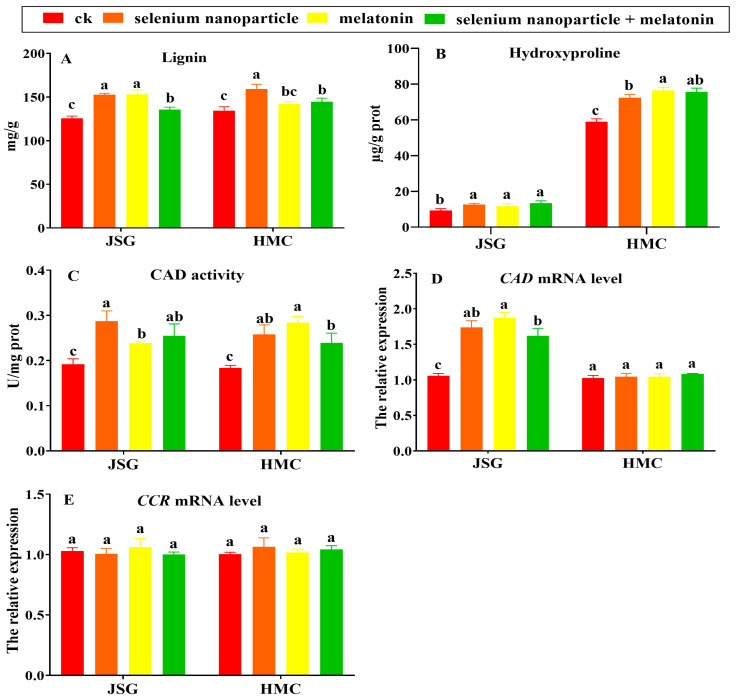
Effects of selenium nanoparticles and melatonin on lignin synthesis in two melon cultivars. JSG and HMC refer to melon cultivars. Different letters indicate a significant difference (*p* < 0.05) between treatments. (**A**): Lignin content, (**B**): Hydroxyproline content, (**C**): CAD activity, (**D**): *CAD* mRNA level, (**E**): *CCR* mRNA level.

The effects of selenium nanoparticles, melatonin, and selenium nanoparticle + melatonin combination on lignin synthesis in melon are shown in Figure 3. Compared with controls, the selenium nanoparticles and selenium nanoparticles + melatonin significantly increased the lignin level in JSG leaves by 22% and 8%, respectively, and in HMC leaves by 18% and 8%, respectively (Figure 3A). The three treatments significantly increased the hydroxyproline level by 25–44% in JSG leaves and by 23–30% in HMC leaves compared with controls (Figure 3B). Compared with controls, selenium nanoparticles, melatonin, and the selenium nanoparticle + melatonin combination significantly increased CAD activities in JSG leaves by 50%, 24%, and 33%, respectively, and in HMC leaves by 40%, 54%, and 30%, respectively (Figure 3C). The three treatments significantly increased the *CAD* mRNA level in JSG leaves by 53–77% compared with controls (Figure 3D). The selenium nanoparticle, melatonin, and selenium nanoparticle + melatonin combination groups had no significant effects on *CCR* expression in either cultivar’s leaves compared with controls (Figure 3E).

#### 2.4.2. Polyamine Metabolism after Selenium Nanoparticle and Melatonin Treatments

**Figure 4:**
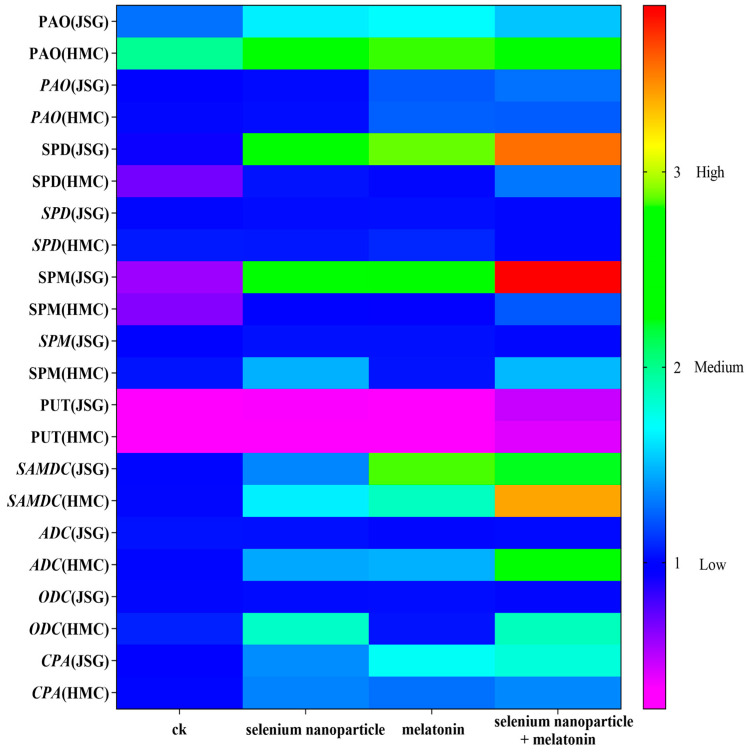
Effects of selenium nanoparticles and melatonin on polyamine metabolites in two melon cultivars. JSG and HMC refer to melon cultivars.

The effects of selenium nanoparticles, melatonin, and selenium nanoparticles + melatonin on polyamine metabolism in melon plants are shown in Figure 4. Compared with controls, the selenium nanoparticles and melatonin significantly increased PAO activity in JSG leaves by 26% and 23%, respectively, and in HMC leaves by 46% and 51%, respectively. The melatonin and selenium nanoparticle + melatonin combination treatments significantly increased the *PAO* mRNA level in JSG leaves by 22–29% and in HMC leaves by 24–23% compared with controls. The three treatments significantly increased the SPD level in JSG leaves by 202–288% and in HMC leaves by 39–80% compared with controls. Compared with controls, the selenium nanoparticle, melatonin, and selenium nanoparticle + melatonin combination treatments had no significant effects on the expression level of *SPD* in JSG and HMC leaves. The three treatments significantly increased SPM by 334–538% in JSG leaves and by 39–83% in HMC leaves compared with controls. Compared with controls, the selenium nanoparticle and selenium nanoparticle + melatonin combination treatments significantly up-regulated the *SPM* mRNA level in HMC leaves by 41% and 46%, respectively. The three treatments significantly increased PUT by 35–96% in JSG leaves and by 16–53% in HMC leaves compared with controls. The selenium nanoparticle, melatonin, and selenium nanoparticle + melatonin combination groups significantly increased the *SAMDC* mRNA level in JSG leaves by 38–180% and in HMC leaves by 64–229% compared with controls. Compared with controls, the three treatments significantly increased the expression level of *ADC* in HMC by 45–136%. Compared with controls, the selenium nanoparticles and selenium nanoparticle + melatonin combination significantly increased the *ODC* transcription level in HMC leaves by 71% and 73%, respectively. The three treatments significantly up-regulated the *CPA* expression in both cultivars by 31–78% compared with controls.

#### 2.4.3. Effects of Selenium Nanoparticles and Melatonin on Phenylpropane Metabolism

**Figure 5:**
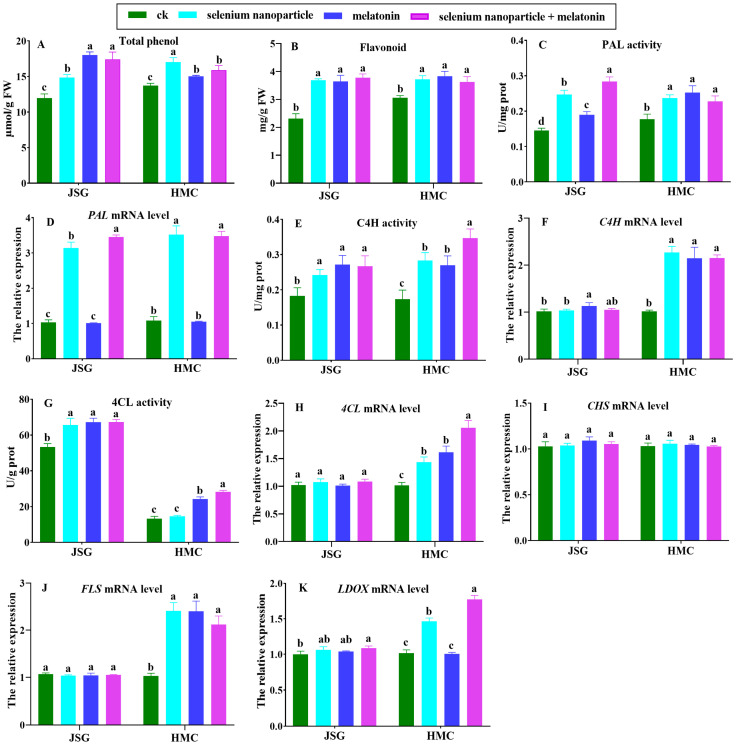
Effects of selenium nanoparticles and melatonin on phenylpropane metabolites in two melon cultivars. JSG and HMC refer to melon cultivars. Different letters indicate a significant difference (*p* < 0.05) between treatments. (**A**): Total phenol content, (**B**): Flavonoid content, (**C**): PAL activity, (**D**): *PAL* mRNA level, (**E**): C4H activity, (**F**): *C4H* mRNA level, (**G**): 4CL activity, (**H**): *4CL* mRNA level, (**I**): *CHS* mRNA level, (**J**): *FLS* mRNA level, (**K**): *LDOX* mRNA level.

The effects of selenium nanoparticles, melatonin, and the selenium nanoparticle + melatonin combination on phenylpropane metabolism in melon are shown in Figure 5. Compared with controls, the three treatments significantly increased total phenols in JSG leaves by 24–51% and in HMC leaves by 10–24% (Figure 5A). The selenium nanoparticle, melatonin, and selenium nanoparticle + melatonin combination groups significantly increased the flavonoid level in both cultivars by 19–64% compared with controls (Figure 5B). The three treatments significantly increased the PAL activity in JSG leaves by 31–95% and in HMC leaves by 29–43% compared with controls (Figure 5C). Compared with controls, the selenium nanoparticle and selenium nanoparticle + melatonin combination treatments significantly increased the *PAL* mRNA level in JSG leaves by 205% and 235%, respectively, and in HMC leaves by 226% and 222%, respectively (Figure 5D). The three treatments significantly increased the C4H activity in both cultivars by 32–100% compared with controls (Figure 5E). The selenium nanoparticle, melatonin, and selenium nanoparticle + melatonin combination groups significantly up-regulated the *C4H* expression in HMC leaves by 111–123% compared with controls (Figure 5F). Compared with controls, the melatonin and selenium nanoparticle + melatonin combination significantly increased 4CL activity by 26% and 26%, respectively, in JSG leaves and by 83% and 113%, respectively, in HMC leaves (Figure 5G). The three treatments significantly increased *4CL* expression in HMC leaves by 41–102% compared with controls (Figure 5H). The selenium nanoparticles, melatonin, and selenium nanoparticle + melatonin combination had no significant effects on the *CHS* expression in either cultivar (Figure 5I), but the three treatments significantly increased the *FLS* transcription level by 107–135% in HMC compared with controls (Figure 5J). Compared with controls, the selenium nanoparticle and selenium nanoparticle + melatonin combination groups significantly up-regulated the *LDOX* expression in HMC leaves by 44% and 75%, respectively (Figure 5K).

### 2.5. Effects of Selenium Nanoparticle and Melatonin Treatment on Antioxidant Capacities

**Figure 6:**
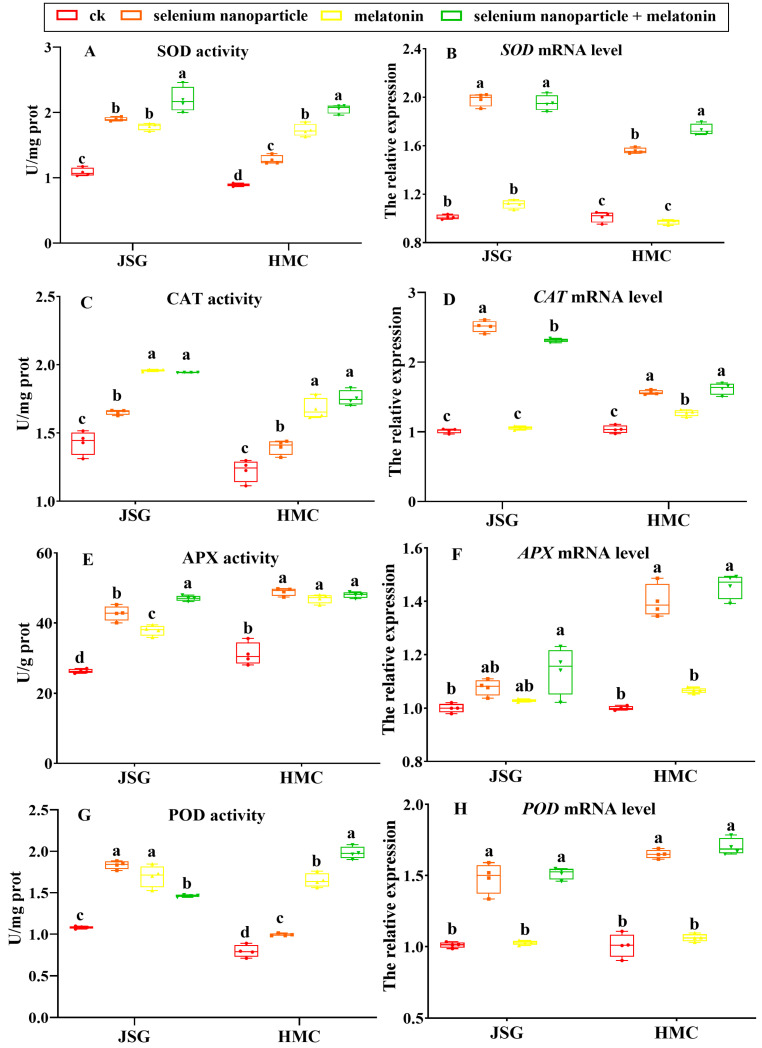
Effects of selenium nanoparticles and melatonin on the antioxidant capacities in two melon cultivars. JSG and HMC refer to melon cultivars. Different letters indicate a significant difference (*p* < 0.05) between treatments. (**A**): SOD activity, (**B**): *SOD* mRNA level, (**C**): CAT activity, (**D**): *CAT* mRNA level, (**E**): APX activity, (**F**): *APX* mRNA level, (**G**): POD activity, (**H**): *POD* mRNA level.

The effects of selenium nanoparticle, melatonin, and selenium nanoparticle + melatonin combination groups on the antioxidant capacities of melon are shown in Figure 6. Compared with controls, the SOD activity after the three treatments significantly increased by 43–130% in the two cultivars (Figure 6A). The selenium nanoparticle and selenium nanoparticle + melatonin combination groups significantly increased the *SOD* mRNA level in JSG leaves by 93–96% and in HMC leaves by 54–71% compared with controls (Figure 6B). The three treatments significantly increased the CAT activity in both cultivars by 14–43% compared with controls (Figure 6C). Compared with controls, the selenium nanoparticle and selenium nanoparticle + melatonin combination treatments significantly up-regulated the *CAT* expression in JSG leaves by 148–128% and in HMC leaves by 51–56% (Figure 6D). The three treatments significantly increased the APX activity in both cultivars by 44–79% compared with controls (Figure 6E). The selenium nanoparticle + melatonin combination group significantly enhanced the *APX* transcription level in the two cultivars by 14–46% compared with controls (Figure 6F). The POD activity after the three treatments increased significantly by 25–149% in the two cultivars compared with controls (Figure 6G). Compared with controls, the selenium nanoparticle and selenium nanoparticle + melatonin combination treatments significantly increased the *POD* mRNA level in JSG leaves by 46–50% and in HMC leaves by 64–69% (Figure 6H).

The effects of selenium nanoparticles, melatonin, and the selenium nanoparticle + melatonin combination on ROS and lipid peroxidation in melon are shown in Figure S2. Compared with controls, the three treatments had no significant effects on the H_2_O_2_ (Figure S2A) and MDA (Figure S2B) contents in JSG and HMC leaves. The selenium nanoparticles, melatonin, and selenium nanoparticle + melatonin combination significantly increased GSH by 12–47% in both cultivars compared with controls (Figure S2C). The proline level in both cultivars after the three treatments were significantly increased by 18–141% compared with controls (Figure S2D). Compared with controls, the scavenging ability of DPPH after the selenium nanoparticle, melatonin, and selenium nanoparticle + melatonin combination treatments significantly increased by 19–38% in the two cultivars (Figure S2E). These results indicated that selenium nanoparticles and melatonin increased the GSH level, proline level, and DPPH scavenging ability, whereas the treatments decreased the ROS level in melon leaves.

## 3. Discussion

Bio-stimulants are used to enhance the growth of plants, whose demand increases 12% per year on the global market [34]. In this study, bio-stimulants had certain promotional effects on the biomasses of melon seedlings, which were related to the increased production of IAA after selenium nanoparticle and melatonin treatments. Exogenous melatonin enhances immunity to the bacterial pathogen *Pseudomonas syringae* through SA [35]. It also promotes the production of sugar, resulting in an increasing SA content [36]. Melatonin plays roles in plant innate immunity against pathogens through SA/JA pathways, and the resulting up-regulation of the gene encoding SA biosynthetic heteromeric acid synthetase (*ICS1*) leads to an increased SA content [37]. The resistance of plants to biological stresses, including crop resistance to fungi, is induced by melatonin through endogenous hormones [38]. After selenium-containing treatments, the level of the defense hormone JA, which induces sulfur assimilation and GSH biosynthesis gene expression, and those of the defense genes related to SA synthesis increase [39]. The selenium nanoparticles and melatonin had no effect on the SA and JA contents in melon seedlings, which was related to the experimental conditions in which melon seedlings were under abiotic stress.

**Figure 7:**
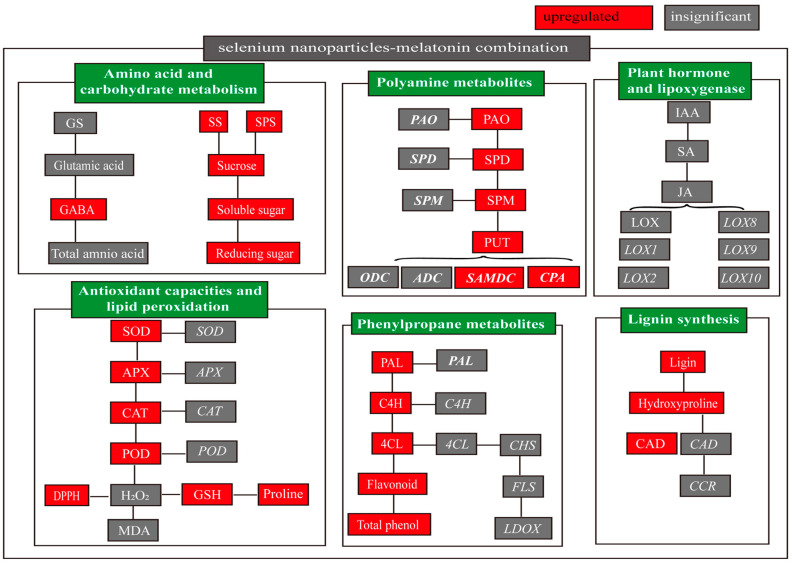
The mechanism of selenium nanoparticles and melatonin regulated carbohydrate, polyamines, and antioxidant capacity of melon plant. Red indicates upregulation, while gray indicates insignificance.

The mechanisms by which selenium nanoparticles and melatonin regulate carbohydrate and polyamine in melon are illustrated in Figure 7. The external stress increases LOX activity, accelerates the oxidation of unsaturated fatty acids catalyzed by LOX, and increases the MDA content [40]. Melatonin inhibits LOX activity and decreases *PbLOX1* and *PbLOX2* mRNA levels [41]. Proline, sugars, and free amino acids are bio-soluble solutes that protect plants from stress through osmotic regulation, ROS clearance, and plasma membrane integrity, and exogenous melatonin increases the selenium-induced contents of proline, free amino acids, and soluble sugars [42]. This was consistent with the selenium nanoparticle, melatonin, and selenium nanoparticle + melatonin combination treatments, which increased levels of primary metabolites, such as melon sugars and amino acids, in the present study. Melatonin improves the activities of sucrose-metabolism-related enzymes, hydrolyzing a large amount of sucrose into glucose and fructose. The increased soluble sugar and antioxidant enzyme activities lead to a greater stress resistance in grape seedlings and increase adaptability to environmental changes [43]. These findings are consistent with the results of the present study. The selenium nanoparticles and melatonin, as bio-stimulants, promoted the primary metabolic abilities of soluble sugar and amino acids in melon seedlings, providing a basis for the utilization of selenium nanoparticles and melatonin in melon cultivation.

Melatonin increases the lignification degree of tea by altering the expression levels of enzymes involved in the lignin synthesis pathway [44]. The hydroxyproline in plant cell walls plays important roles in plant growth, development, and defense [45]. This same trend was shown in the current study, with selenium nanoparticles, melatonin, and selenium nanoparticles + melatonin increasing levels of secondary metabolites, such as lignin and phenylpropane, in melon. Melatonin is involved in signaling in plants through its receptors and downstream signal transduction pathways [46]. The applications of 50 and 100 μmol·L^−1^ melatonin on leaves have greater effects on fruit quality than on leaf quality, and this significantly increases the phenolic content (including total phenols and flavonoids) [47]. Melatonin mitigates heat stress by increasing levels of soy phenols, flavonoids, prolines, and endogenous melatonin and polyamine [48]. In our study, selenium nanoparticles, melatonin, and selenium nanoparticles + melatonin increased SPM, SPD, PUT, and mRNA levels. Selenite up-regulates the expression of genes related to the biosynthesis of phenylpropane compounds and the activities of related enzymes, such as PAL, C4H, chalcone synthase, chalcone isomerase, and CAD [49]. Melatonin enhances the postharvest disease resistance of blueberry fruit by regulating phenylpropane metabolism (PAL, C4H, 4CL, and CAD activities) and mRNA levels [50]. This is consistent with the results of selenium nanoparticle, melatonin, and selenium nanoparticle + melatonin treatments used in this study.

Melatonin plays different roles in plants, enhancing the activities of a variety of antioxidant enzymes, including SOD, CAT, and POD, and controlling ROS, as well as other free radicals, present in plant cells [51]. The combination treatment of melatonin and Na_2_SeO_3_ enhances the resistance of fruit to gray mold by increasing the activities of SOD, POD, and CAT, as well as the expression levels of disease-related genes [52]. Melatonin–selenium nanoparticles increase the activity levels of antioxidant enzymes, such as SOD, POD, CAT, APX, and GSH, and decrease the contents of MDA and H_2_O_2_ [53]. The application of selenium nanoparticles significantly increased the antioxidant capacity and decreased the MDA content. Compared with soil selenium applications, foliar selenium applications are efficient and safe, resulting in the delayed development of plaques on sunflower leaves. The application method leads to the absorption of selenite and efficient conversion to selenomethionine (80%) and selenium-containing proteins, which not only have enzymatic functions but also act as antioxidants through the direct scavenging of free radicals [54]. In future research, the effects of selenium nanoparticles and melatonin on the fruit quality of melon should be determined using carotenoids or other quality indicators. Additionally, the effects of bio-stimulants on the incidence of melon disease under biotic stress should be explored to provide evidence for the safe cultivation of melon.

## 4. Materials and Methods

### 4.1. Experimental Design

JSG and HMC melons were used as the study materials. The seeds of JSG and HMC were provided by the Hami Melon Research Center of the Xinjiang Academy of Agricultural Sciences (Urumqi, China). Full seeds were soaked, germinated, and planted in plastic pots (30 cm × 30 cm), with two plants per pot. The preparation and characterization of selenium nanoparticles has been reported previously [55]. The concentrations of selenium nanoparticles [33] and melatonin [56] used in this study were based on previous studies by our research group. When the seedlings grew to the 3–4 leaf stage, the leaves were spray-treated. Three treatments were established. There was a control (CK), as well as 5 mg·L^−1^ selenium nanoparticles, 5 mg·L^−1^ melatonin and 5 mg·L^−1^ selenium nanoparticles + melatonin combination groups, and each treatment was repeated three times. The water (control), selenium nanoparticles (5.0 mg·L^−1^), melatonin (5.0 mg·L^−1^), and selenium nanoparticle + melatonin combination (5.0 mg·L^−1^) were sprayed on the seedling leaves. All the melon seedlings were placed outdoors, at 20 °C to 30 °C. The foliar spraying was carried out from 9:00 to 10:00 AM on windless and sunny days, and the spraying was based on no dripping from the foliar surface. After 7 days, melon leaves were collected, frozen in liquid nitrogen, and stored at −80 °C. The plant heights and stem diameters of melon seedlings were measured using a ruler and a vernier caliper, respectively. The fresh weights of the stems were measured after the leaves were collected, and the dry weights of the stems were measured after drying.

### 4.2. Determination of Lipid Peroxidation Products

The leaves were ground after being frozen in liquid nitrogen. Then, 0.1 g of each powdered sample was placed independently in a tube containing 1 mL extraction solution in accordance with the instructions of the appropriate test kit. Samples were homogenized and centrifuged at 12,000× *g* at 4 °C for 10 min for H_2_O_2_ and DPPH determinations, or at 4000× *g* at 4 °C for 10 min for MDA, proline, and reducing glutathione determinations. The obtained supernatants were treated using kits for H_2_O_2_ (Kit number: A064-1-1), MDA (Kit number: MDA, A003-1-1), proline (Kit number: A107-1-1), and DPPH (Kit number: A153-1-1) from Nanjing Jiancheng Bioengineering Institute (Nanjing, China). Reducing glutathione (GSH) was determined using a kit (Kit number: E-BC-K030-M, Elabscience Biotechnology Co., Ltd., Wuhan, China). The H_2_O_2_, MDA, proline, and DPPH levels were determined using a UV–visible spectrophotometer (Shanghai Shunyu Hengping Instrument Co., Ltd., Shanghai, China). GSH was determined using a multi-scan spectroscopic microporous plate spectrophotometer (Bio Tek Instruments Inc., Winooski, VT, USA). Obtained liquid samples were used to measure absorbance at 405 nm for H_2_O_2_, at 517 nm for DPPH, at 532 nm for MDA, at 520 nm for proline, and at 405 nm for GSH. The standard curve values for GSH, proline, and DPPH are shown in Table S1.

### 4.3. Determinations of Plant Primary and Secondary Metabolites

The SOD (Kit number: A001-3), CAT (Kit number: A007-1-1), APX (Kit number: A123-1-1), peroxidase (POD, Kit number: A084-3), phenylalanine ammonia lyase (PAL, Kit number: A137-1-1), glutamine synthetase (GS, Kit number: A047-1-1), total phenols (Kit number: A143-1-1), flavonoids (Kit number: A142-1-1), proteins (Kit number: A045-4), total amino acids (Kit number: A026-1-1), glutamic acid (Kit number: A074-1-1), lipoxygenase (LOX, H550-1), soluble sugar (Kit number: A145-1-1), sucrose (Kit number: A099-1-1), sucrose synthetase (SS, Kit number: A097-1-1), and sucrose phosphate synthetase (SPS, Kit number: A098-1-1) kits were procured from Nanjing Jiancheng Bioengineering Institute. The reducing sugar (Kit number: BC0235), trans-cinnamate 4-hydroxylase (C4H, Kit number: BC4080), co-A ligase (4CL, Kit number: BC4220), cinnamyl alcohol dehydrogenase (CAD, Kit number: BC4170), lignin (Kit number: BC4200), and hydroxyproline (Kit number: BC0255) kits were purchased from Solarbio Science and Technology Company Ltd. (Beijing, China). The polyamine oxidase (PAO) kit was procured from Shanghai Enzyme Linked Biotechnology Co., Ltd. (Shanghai, China).

The main steps for determining plant primary and secondary metabolite levels were as follows: melon leaves were ground with a pestle and a mortar containing liquid nitrogen. In total, 0.1 g of each powdered sample was placed in a tube containing 1 mL of the appropriate extraction solution in accordance with the instructions of an appropriate kit. Then, samples were homogenized and centrifuged at 800× *g* at 4 °C for 10 min for SOD, CAT, and POD; at 10,000× *g* at 4 °C for 10 min for APX and flavonoids, at 12,000× *g* at 4 °C for 10 min for PAL, at 4000× *g* at 4 °C for 10 min for GS and total phenols, at 2500× *g* at 4 °C for 10 min for proteins, at 3500× *g* at 4 °C for 10 min for total amino acids, at 2500× *g* at 4 °C for 10 min for glutamic acid, at 2000× *g* at 4 °C for 20 min for LOX, at 4000× *g* at 4 °C for 10 min for soluble sugar, at 8000× *g* at 4 °C for 10 min for SS and SPS, at 8000× *g* at 4 °C for 10 min for reducing sugar, at 8000× *g* for 10 min for lignin, at 16,000× *g* for 5 min for hydroxyproline, at 12,000× *g* at 4 °C for 10 min for C4H, at 8000× *g* at 4 °C for 10 min for 4CL, at 10,000× *g* at 4 °C for 10 min for CAD, and at 10,000× *g* at 4 °C for 10 min for PAO. The SOD, CAT, POD, LOX, reducing sugar, and PAO levels were determined from obtained supernatants using the appropriate kit and a multi-scan spectrum microplate spectrophotometer, and APX, PAL, SS, SPS, flavonoids, GS, total phenols, proteins, total amino acids, glutamic acid, soluble sugar, reducing sugar, lignin, hydroxyproline, C4H, 4CL, and CAD levels were obtained using appropriate kits and a UV–vis spectrophotometer (Shanghai Shunyu Hengping Instruments Co., Ltd., Shanghai, China) following the manufacturer’s instructions. Obtained liquid samples were used to measure absorbance at 450 nm for SOD, at 405 nm for CAT, at 420 nm for POD, at 290 nm for APX and PAL, at 540 nm for GS, at 760 nm for total phenols, at 502 nm for flavonoids, at 562 nm for proteins, at 650 nm for total amino acids, at 340 nm for glutamic acid, at 450 nm for LOX, at 620 nm for soluble sugar, at 480 nm for SS and SPS, at 540 nm for reducing sugar, at 280 nm for lignin, at 560 nm for hydroxyproline, at 340 nm for C4H, at 333 nm for 4CL, at 340 nm for CAD, and at 450 nm for PAO. The standard curve values for POD, total phenols, flavonoids, total amino acids, glutamic acid, LOX, soluble sugar, SS, SPS, reducing sugar, and PAO are shown in Table S1.

### 4.4. Ultra Performance Liquid Chromatography Tandem Mass Spectrometry (UPLC–MS/MS) Analyses of Polyamines, γ-Aminobutyric Acid (GABA), and Plant Hormones

The samples were homogenized in 3.0 mL acetonitrile solution (8:2, *v*/*v*) and centrifuged at 4500× *g* at 4 °C for 10 min. The detection conditions for polyamines were as follows: HILIC column (100 mm × 2.1 mm, 1.7 µm), mobile phase: (A) 5 mmol/L ammonium acetate acetonitrile solution and (B) 0.1% formic acid solution; gradient elution: 0–1.0 min, 80% A; 1.0–1.5 min, 80–40% A; 1.5–6.0 min, 40% A; 6.0–6.5 min, 40–80% A; 6.5–9.5 min, 80% A; injection volume: 2 µL; flow rate: 0.3 mL/min; column temperature: 30 °C. In positive ion mode, the ion source temperature was 150 °C, and the solvent temperature was 350 °C. The N_2_ flow rate of the cone hole was 650 L/h, and the N_2_ flow rate was 250 L/h. The mass spectrum conditions of putrescine (PUT), spermine (SPM), spermidine (SPD), GABA, jasmonic acid (JA), salicylic acid (SA), and indoleacetic acid (IAA) are shown in Table S2. The samples were homogenized in 1.0 mL methanol/water/formic acid (15:4:1, *v*/*v*/*v*) and centrifuged at 4 °C for 5 min at 16,000× *g*. The chromatographic conditions of the three plant hormones have been published previously [33]. The chromatographic conditions for melatonin were as follows: chromatographic column: ACQUITY UPLC BEH C18 (1.7 μm, 2.1 mm × 100 mm); mobile phase A: 0.1% (*v*/*v*) formic acid aqueous solution; mobile phase B: acetonitrile; injection volume 1 μL; flow rate of 0.2 mL/min. The mass spectrometry conditions were as follows: electrospray ionization (ESI^+^); multiple reaction monitoring mode detection; detected ion pair: *m*/*z* 233→174. The other mass spectrometry tuning parameters were the same as those for plant hormones. The standard curve of melatonin was y = 318917x + 914976, R^2^ = 0.9967. The mass spectra of the standard for melatonin is shown in Figure S3.

### 4.5. RT-qPCR Analysis

Total RNA was extracted using kits and reverse-transcribed into cDNA (Transgen Biotech Company Ltd., Beijing, China). The cDNA synthesis system is shown in Table S3. Primer sequences for target and actin control genes are shown in Table S4. Relative mRNA levels were calculated using 2^−ΔΔCt^.

### 4.6. Statistical Analyses

SPSS (version 26 SPSS Inc., Chicago, IL, USA) was used for the one-way analysis of variance (ANOVA). Tukey’s tests were used for multiple comparisons to determine significant differences (*p* < 0.05). Graph Pad Prism software (version 8.02; San Diego, CA, USA) was used to calculate data statistics.

## 5. Conclusions

Selenium nanoparticle and melatonin foliar treatments facilitated melon seedling growth by regulating primary, secondary, and oxidative-stress metabolism. Independent applications of 5 mg·L^−1^ selenium nanoparticles, melatonin, and the selenium nanoparticle + melatonin combination increased stem dry weights of the JSG and HMC melon cultivars. Compared with control, selenium nanoparticles, melatonin, and the selenium nanoparticle + melatonin combination increased soluble sugar, sucrose, and SPS contents. Additionally, selenium nanoparticles, melatonin, and selenium nanoparticles + melatonin enhanced GABA, hydroxyproline, SPM, SPD, PUT, total phenols, flavonoids, GSH, and proline contents, as well as PAL, C4H, SOD, CAT, APX, and POD activities.

## Data Availability

Data are available upon request from the corresponding author.

## Supplementary Materials

The following supporting information can be downloaded at: https://www.mdpi.com/article/10.3390/ijms25147830/s1.
