# Supplementary material for: Selenium Nanoparticle and Melatonin Treatments Improve Melon Seedling Growth by Regulating Carbohydrate and Polyamine
Source: Int J Mol Sci. 2024 Jul 17;25(14):7830. doi: 10.3390/ijms25147830 (PMC11276989; doi:10.3390/ijms25147830)
Supplement: Supplementary file 1 [file ijms-25-07830-s001.zip › ijms-3100743-supplementary.pdf]

Supporting information

Selenium nanoparticle and melatonin treatments improve melon seedling growth by regulating carbohydrate and polyamine

Lu Kang[a, b, c], Yujiao Jia[a], Yangliu Wu[d], Hejiang Liu[c], Duoyong Zhao[c], Yanjun Ju[c], Canping Pan[a*], Jiefei Mao[b*]

[a] Key Laboratory of National Forestry and Grassland Administration on Pest Chemical Control & Innovation Center of Pesticide Research, College of Science, China Agricultural University, Beijing, 100193, China

[b] State Key Laboratory of Desert and Oasis Ecology, Key Laboratory of Ecological Safety and Sustainable Development in Arid Lands, Xinjiang Institute of Ecology and Geography, Chinese Academy of Sciences, Urumqi 830011, China

[c] Institute of Agricultural Quality Standards and Testing Technology, Xinjiang Academy of Agricultural Sciences, Urumqi 830091, China

[d] School of Biological Science and Technology, University of Jinan, Jinan 250022, China

Corresponding author: Canping Pan

[*]**E-mail:** canpingp@cau.edu.cn

Fax: +86-10-62733620; Tele: +86-10-62731978

Address: 2 Yuanmingyuan Western Road, Haidian District, Beijing 100193, China.

Corresponding author: Jiefei Mao

[*]**E-mail:** mjf@ms.xjb.ac.cn

Tele: +86-991-7827370

Address: 818 Beijing South Road, New urban District, Urumqi 830000, Xinjiang Uygur Autonomous Region, China.

Table captions

Table S1. Standard curve for determining parameters using kit

Table S2. UPLC-MS/MS conditions for the detection of jasmonic acid, salicylic acid, indoleacetic acid, polyamine and γ-aminobutyric acid

Table S3. cDNA synthesis system

Table S4. Primers used in RT-qPCR analysis for gene expression.

Figure captions

Figure S1. Mass spectrum of standard curve for melatonin determination using UPLC-MS/MS.

Figure S2. Effects of selenium nanoparticle and melatonin on plant heights, stem diameters, fresh and dry stem weight of melon.

Figure S3. Effects of selenium nanoparticle and melatonin on ROS and lipid peroxidation in two melon cultivars.

Table S1. Standard curve for determining parameters using kit

Number	Parameter	Standard curve	R^2
1	POD	$y = 0.1554\ln(x) + 0.7013$	$R^2 = 0.9521$
2	Flavonoids	$y = 9.1629x + 0.0032$	$R^2 = 0.9966$
3	Total phenols	$y = 0.0009x + 0.0175$	$R^2 = 0.9975$
4	Total amino acids	$y = 0.0068x + 0.0065$	$R^2 = 0.9940$
5	Glutamic acid	$y = 0.6653x + 0.00004$	$R^2 = 0.9946$
6	LOX	$y = -0.0176x^3 + 0.241x^2 - 0.9644x + 1.3498$	$R^2 = 0.9999$
7	Soluble sugar	$y = 0.005x + 0.0098$	$R^2 = 0.9973$
8	SS	$y = 0.0843x + 0.0261$	$R^2 = 0.9951$
9	SPS	$y = 0.0912x - 0.0042$	$R^2 = 0.9964$
10	Reducing sugar	$y = 0.0063x + 0.0075$	$R^2 = 0.9952$
11	PAO	$y = 0.0008x + 0.0654$	$R^2 = 0.9985$
12	DPPH	$y = 3.1881x + 0.8076$	$R^2 = 0.9969$
13	Proline	$y = 0.0683x - 0.0035$	$R^2 = 0.9938$
14	GSH	$y = 0.0038x + 0.0025$	$R^2 = 0.9931$

Table S2. UPLC-MS/MS conditions for the detection of jasmonic acid, salicylic acid, indoleacetic acid, polyamine and γ-aminobutyric acid.

Name	Formula	Mass	Ion Transition	Cone(V)	Collision(V)
Jasmonic acid	$C_{12}H_{18}O_3$	209.0	59.2,165.0	29.0	14.0
				29.0	15.0
Salicylic acid	$C_7H_6O_3$	137.1	93.1, 65.0	20.0	15.0
				20.0	29.0
Indoleacetic acid	$C_{10}H_9NO_2$	171.2	127.2, 99.0	27.0	13.0
				27.0	16.0
Putrescine	$C_4H_{12}N_2$	89.2	72.4,55.0	45.0	18.0
				45.0	7.0
Spermine	$C_{10}H_{26}N_4$	203.2	84.3,112.3	69.0	18.0
				69.0	30.0
Spermidine	$C_7H_{19}N_3$	146.2	129.1,112.4	46.0	6.0
				46.0	12.0
γ-aminobutyric acid	$C_4H_9NO_2$	104.0	87.0,69.0	33.0	13.0
				33.0	46.0

Table S3. The cDNA synthesis system.

Component	Volume
Total RNA	7 μL
Anchored Oligo $(dT)_{18}$ Primer (0.5 μg/μL)	1 μL
2×TS Reaction mix	10 μL
TranScript® RT/RI Enzyme Mix	1 μL
gDNA Remover	1 μL
Total volume	20 μL

Table S4. Primers used in RT-qPCR analysis for gene expression.

Gene	Serial number	Primer sequence
Actin	XM_008442791.2	5′-CCCTGGTATTGCAGACAGGA-3′<br>5′-ACATCTGCTGGAAGGTGCTT-3′
SOD	XM_008452515.2	5′-ACGGGTAATGTTTCTGGTCTCA-3′<br>5′-TGTTGTTTTCCAGCAGGGTT-3′
CAT	XM_008466617.2	5′-GCCATTCTATCGTCGTATCCA-3′<br>5′-ATCACAGTCACGCCACTCAGG-3′
APX	NM_001297449.1	5′-AAGCAACAGTTCCCCGTCCT-3′<br>5′-TCAGAGCCCTTGGTAGCATCAG-3′
POD	XM_051080054.1	5′-GGCTTTGTTCTCTTGTCTGTGG-3′<br>5′-AGTACGTGAGGAATGATCGTGGT-3′
GLU	NM_001328463.1	5′-CGTCCTTCAGCGATCTTGGT-3′<br>5′-ACGCCATTGCTGGTTGAGAT-3′
CHT1	NM_001328464.1	5′-TGCCGTACTTGGAGCTCTTC-3′<br>5′-TCGGTAACAGCTTCGCACTT-3′
CHT2	NM_001328440.1	5′-ATTCGACGGCGTGGACATTA-3′<br>5′-GAGACGAACACTGTGGAGCA-3′
PAO	XM_008453623.2	5′-ACGTTGGAGTGGGGCTTGA-3′<br>5′-ATGGCTTGTGGCCTTGTGA-3′
SPD	XM_008454883.3	5′-GCTTGGACCATTGTTCCTGCT-3′<br>5′-GGGATTGATTGGATTTTTGAAGTC-3′
SPM	XM_051086969.1	5′-GTTGGCGATGCTGTTGAGTTC-3′<br>5′-TGCTTGGGCTGTCATTCGA-3′

Gene	Serial number	Primer sequence
SAMDC	NM_001328465.1	5′-CATCAAAACTTGCGGCACTAC-3′<br>5′-ATAGCCATCAAGGACAGCAACT-3′
ADC	XM_008462801.3	5′-TGTAGATTATCAGAACCTTTCGGCT-3′<br>5′-TTCCATTCCCAAACACCCATC-3′
ODC	XM_008447089.2	5′-CGTCGTTGGCGTGTCATTT-3′<br>5′-AAGTCGGACTGCCGTTTCG-3′
CPA	XM_051079922.1	5′-AGGGAGGATTTTATTCAACGAGC-3′<br>5′-TTCTCCTGGTAGCCTGGTCC-3′
LOX1	XM_051084704.1	5′-CCATCAACTTATCAGCCATT-3′<br>5′-GTTCGTTCAAGAAGACCAT-3′
LOX2	XM_008456208.3	5′-TAGCACCGAAGGAATCAC-3′<br>5′-AGACAGCACAATAACAGAGT-3′
LOX8	LN713264.1	5′-GGTAACTGGTCGTGGAAT-3′<br>5′-AAGGCAGAGGAATAACAGAA-3′
LOX9	XM_008451331.3	5′-CAGATCCATCTTGTGAAC-3′<br>5′-AGTTGGTAGAGTCATTCC-3′
LOX10	LN713259.1	5′-TGACAGGACAAGGAGTTC-3′<br>5′-CGGTATTGGCAAGAATGTTA-3′
PAL	LN713262.1	5′-ACTCTTCCTTACCTAATACTAAG-3′<br>5′-TTCACCTCATCAAGATGGCTTCC-3′
C4H	XM_008458031.3	5′-AACCACCGTAACCTCACAGATC-3′<br>5′-TTCCTCCAGTGTTCACCATAGA-3′

Gene	Serial number	Primer sequence
4CL	XM_051083764.1	5′-GCAGAAATCGCAAAGCAAGC-3′<br>5′-CATAAACAACCATCGTGAGGAGAAT-3
CHS	XM_008451762.3	5′-TCCTCAAGCTCCTCGGTCTC-3′<br>5′-CTCCTCTGTTGTTCTCCGCTAAA-3′
FLS	XM_008450594.3	5′-CCACAAGTCTGGGAAGATGTC-3′<br>5′-TCATAAGTCAACCTCAGCCG-3′
LDOX	XM_051087074.1	5′-CTTTCTCAGCGTCTCTTAGCC-3′<br>5′-CAGTAACCCAATCACCTTGC-3′
CAD	XM_008457948.3	5′-GAGACGCAAGAAGTATTG-3′<br>5′-ACTCAGGCATCTTACTAC-3′
CCR	XM_051091715.1	5′-TTATTTGGCGTGACGGTCTC-3′<br>5′-TACATCTCAGCCGCTCCTTTC-3′

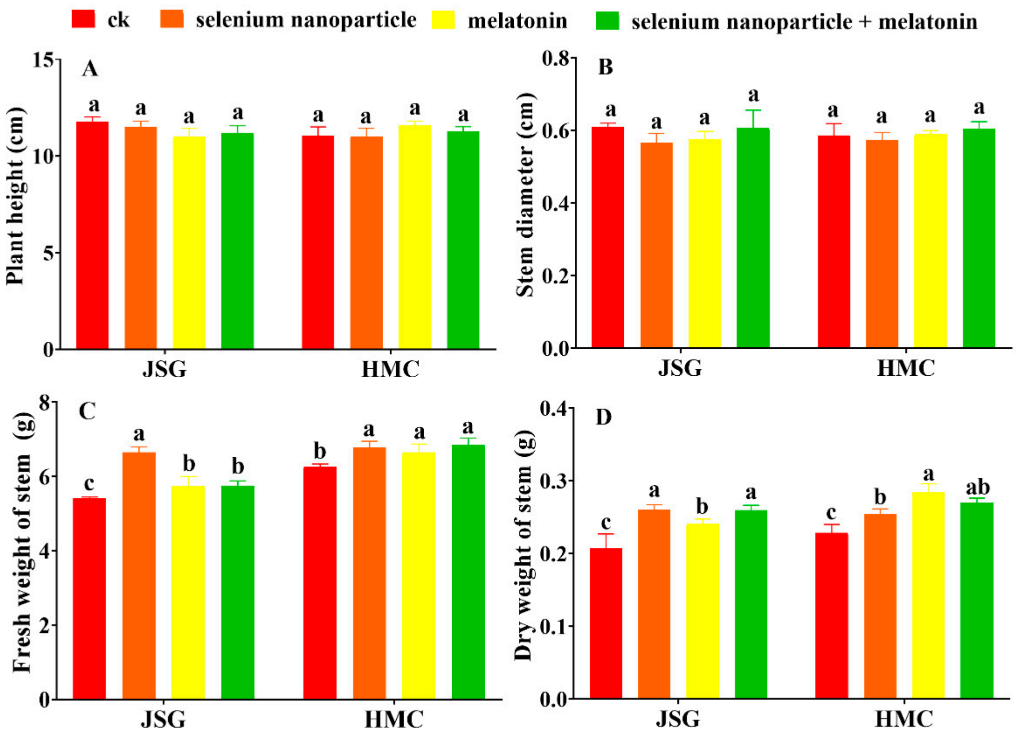

Figure S1. Effects of selenium nanoparticle and melatonin on plant heights, stem diameters, fresh and dry stem weight of melon. JSG and HMC refer to melon cultivars. Different letters indicate a significant difference ($P < 0.05$) between treatments. A: Plant height, B: Stem diameter, C: Fresh weight of stem, D: Dry weight of stem.

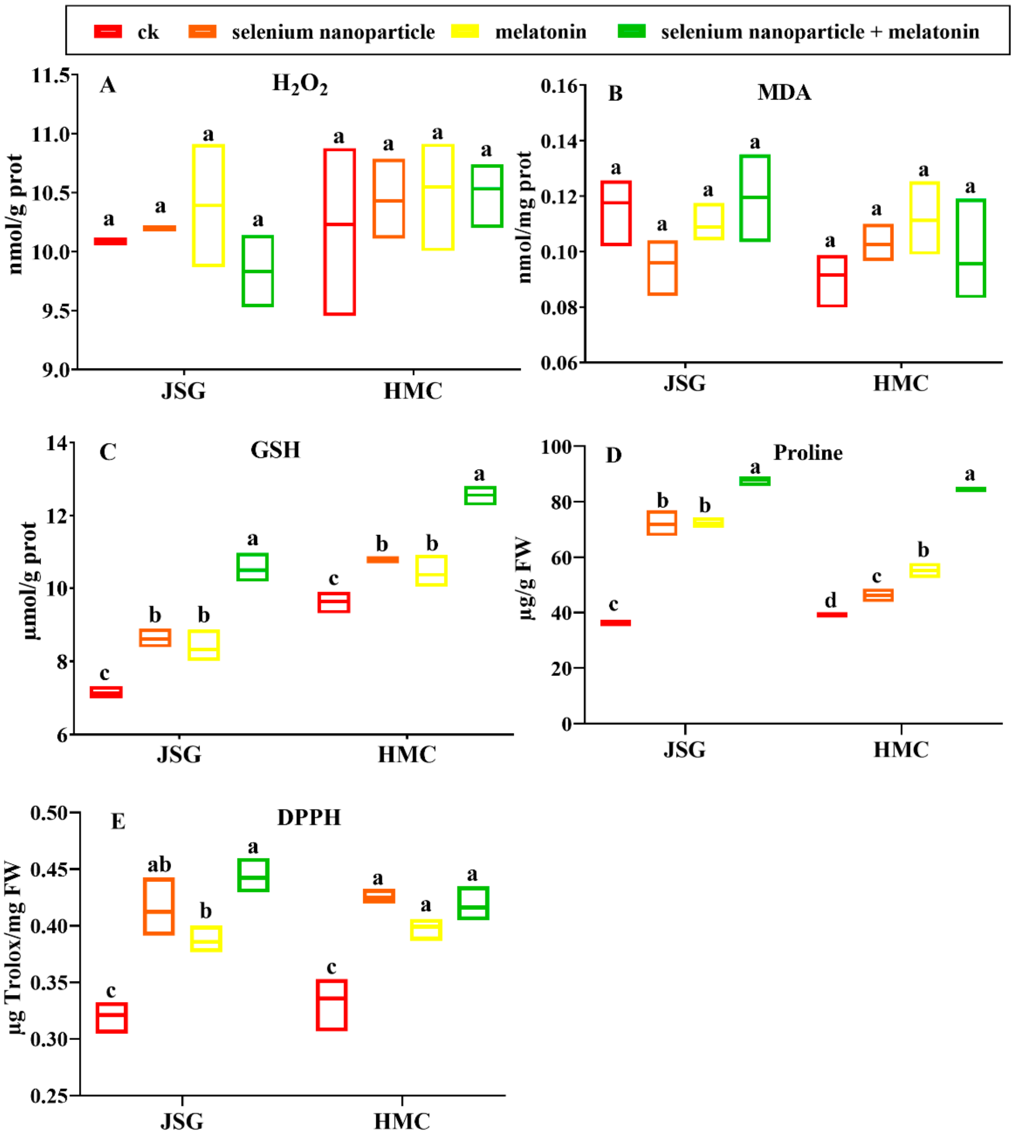

Figure S2. Effects of selenium nanoparticle and melatonin on ROS and lipid peroxidation in two melon cultivars. JSG and HMC refer to melon cultivars. Different letters indicate a significant difference ($P < 0.05$) between treatments. A: H_2O_2 content, B: MDA content, C: GSH content, D: Proline content, E: DPPH.

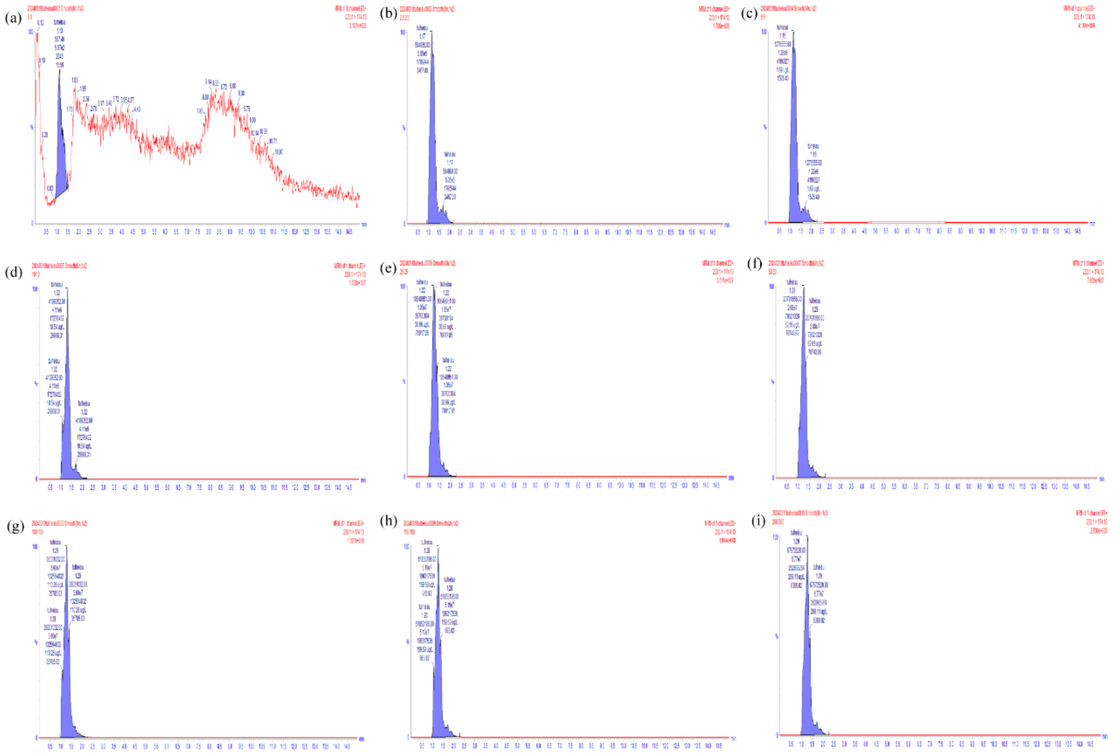

Figure S3. Mass spectrum of standard curve for melatonin determination using UPLC-MS/MS. (a), (b), (c), (d), (e), (f), (g), (h), and (i) represent 0.00, 2.67, 5.33, 10.67, 26.67, 53.34, 106.67, 160.00, and 213.34 μg/L of melatonin mass spectrum, respectively.
